# Timing of Osteoporotic Vertebral Fractures in Lung and Heart Transplantation: A Longitudinal Study

**DOI:** 10.3390/jcm9092941

**Published:** 2020-09-11

**Authors:** Carla Caffarelli, Maria D. Tomai Pitinca, Mario Alessandri, Paolo Cameli, Elena Bargagli, David Bennett, Antonella Fossi, Sonia Bernazzali, Stefano Gonnelli

**Affiliations:** 1 Department of Medicine, Surgery and Neuroscience, University of Siena, 53100 Siena, Italy; dea_to79@yahoo.it (M.D.T.P.); mralessandri@gmail.com (M.A.); gonnelli@unisi.it (S.G.); 2 Respiratory Diseases and Lung Transplantation Unit, University of Siena, 53100 Siena, Italy; paolocameli88@gmail.com (P.C.); bargagli2@gmail.com (E.B.); david.btt@gmail.com (D.B.); a.fossi@ao-siena.toscana.it (A.F.); 3 Department of Cardiac Surgery, University of Siena, 53100 Siena, Italy; s.bernazzali@gmail.com

**Keywords:** osteoporosis, vertebral fracture, lung transplantation, heart transplantation, bone mineral density

## Abstract

Bone loss and bone fractures are common complications after organ transplantation. Many factors contribute to the pathogenesis of transplant osteoporosis, such as bone disease preceding transplantation, immunosuppressive medications, and nutritional and lifestyle factors. This study aimed to assess the incidence of vertebral fractures before and after lung and heart transplantation. This longitudinal study analyzed 213 electronic medical records of patients who underwent lung transplantation (*n* = 128) and heart transplantation (*n* = 85) at Siena University Medical Center between January 2000 and December 2018. In lung and heart transplant recipients, the bone mineral density in the femoral sub-regions show a significant decrease at post-transplantation and at follow up visits. In both lung and heart recipients, we found an increase in the fracture incidence in the first period after transplantation (19.5% vs. 50.4% in lung recipients; 9.6% vs. 25.7% in heart recipients). Moreover, in lung recipients, vertebral fractures were predicted primarily by age, BMD at the femur, and any history of fracture. In heart recipients, vertebral fractures were predicted only by history of fracture. Our study supports the recommendations for pre-transplant osteoporosis screening in patients undergoing lung transplants, and in the first period after transplantation in heart transplant recipients.

## 1. Introduction

Organ transplantation has become an established therapy for many end-stage diseases, including acute and chronic liver failure, end-stage renal disease, end-stage pulmonary disease, and heart failure [1,2]. The number of successful organs transplanted has increased, as well as the survival of the transplant recipients. This has resulted in increased recognition of the long-term complications of transplantation itself, and also complications arising from the action of immunosuppressive drugs [3]. Osteoporosis and fragility fractures are among the complications that have become more prevalent with the increased survival of patients after solid organ transplantation. The prevalence of osteoporosis among candidates expecting various organ transplantations is high; in particular, in subjects awaiting lung transplant, osteoporosis was estimated to range from 35% to 61% [4,5]. Moreover, approximately 8% to 10% of cardiac transplantation candidates have osteoporosis or osteopenia [6]. The main consequence of osteoporosis is bone fractures, which are associated with increased morbidity and mortality rates. Vertebral fractures are the most common type of osteoporotic fracture and have important consequences, including increased risk for subsequent fracture and reduced quality of life [7]. Moreover, in this particular group of patients, increased spine fracture burden was associated with an increase in back pain, spinal deformity, shorter stature, decreased mobility and physical performance, and most importantly, significant decreases in forced inspiratory vital capacity (lung volume) and inspiratory time [8]. The pathogenesis of transplant-associated osteoporosis is multifactorial and involves altered bone metabolism during the pre-transplant period, post-transplant bone loss caused by immunosuppressive therapy and corticosteroids, persistent hyperparathyroidism, and vitamin D deficiency [9,10]. However, it should be kept in mind that patients with end-stage heart or lung disease come to transplantation with different types of underlying bone disease relating to their disease pathogenesis and various pharmacological treatments [2,3,11].

The aim of this longitudinal study was twofold:

1. To evaluate the prevalence of osteoporosis and fragility fractures in a population of adults before and after receiving a heart or lung transplant.
2. To elucidate any factors which may influence fractures in heart or lung transplantation subjects.

## 2. Materials and Methods

**Figure 1:**
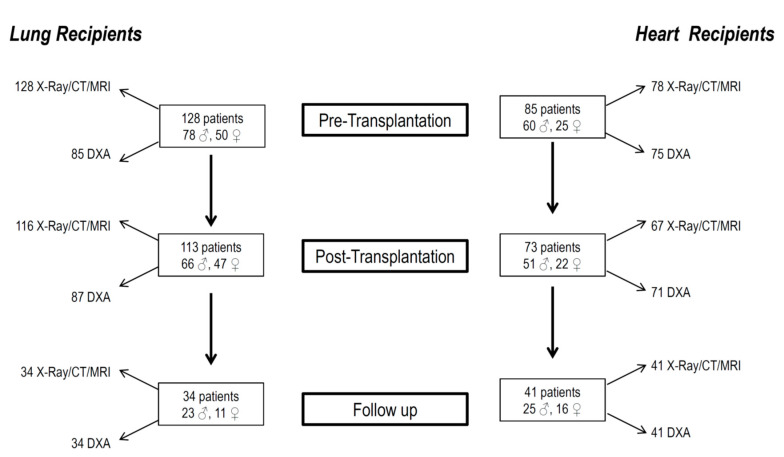
Flowchart showing patient enrollment and follow-up.

This is a retrospective observational study which assessed 213 consecutive cardiac or lung transplant patients (aged ≥ 20 years) at Siena University Medical Center between January 2000 and December 2018 (Figure 1).

One hundred and twenty-eight patients who underwent lung transplants at the Interstitial Lung Diseases of the Respiratory Diseases and Lung Transplant Unit, Department of Medical, Surgical and Neurological Sciences, University Hospital of Siena, were included in the study. Seventy-eight males and 50 females, aged 50.7 ± 11.8 years, made up the sample. These patients had underlying diseases which led to the transplant, including pulmonary fibrosis (67/128 patients, 52.3% of the study population); chronic obstructive pulmonary disease (26/128 patients, 20.3%); cystic fibrosis (22/128 patients, 17.2%); and other pulmonary diseases (13/128 patients, 10.2%).

Eighty-five patients who underwent heart transplants at the Department of Cardiovascular Diseases, University of Siena, were included in the study. They were 60 males and 25 females, aged 51.1 ± 10.4 years. In these latter patients, the underlying diseases included dilated cardiomyopathy (37/85 patients, 43.6% of the study population), ischemic heart disease (30/85 patients, 35.3% of the study population), or other valve disorders and congenital diseases (18/85 patients, 21.1%). In both the lung and heart groups, patients treated with antiosteoporosis drugs (bisphosphonates, raloxifene, denosumab or teriparatide) were excluded.

We selected patients who had undergone an assessment within the 12 months before transplantation (pre-transplantation visit; 5.2 ± 4.3 months), and 6–18 months after transplantation (post-transplantation visit; 11.7 ± 2.2 months). When available, we also evaluated any follow-up visit (4.7 ± 1.9 years).

Information was obtained and analyzed by way of electronic medical records. All patients’ medical information was reviewed carefully for data on age at time of transplant, height, weight, gender, and disease leading to lung or heart transplantation. Information was collected during an osteoporosis visit focused on assessing the history of previous fractures and post-transplantation incident fractures.

All radiographs were examined for the presence of any vertebral fracture according to Genant’s method [12]. Namely, the vertebrae were identified and morphometry was performed from the fourth thoracic vertebra (T4) to the fourth lumbar vertebra (L4) by marking six points in each vertebral body, corresponding to the four corners and the midpoints of the endplates. The anterior (Ha), mid-vertebral (Hm), and posterior (Hp) heights of each vertebra were measured and the three ratios, Ha/Hp, Hm/Hp, and Hp/Hp-below, were calculated [12]. Two of the authors (G-S and C-C) independently performed vertebral morphometry manually. In cases of divergent opinions, consensus was reached by discussion with a radiologist.

Previous Dual-Energy X-Ray Absorptiometry (DXA) scans of the lumbar spine (LS-BMD) and femoral sub-regions (femoral neck (FN-BMD) and total hip (TH-BMD)), radiographs (X-ray) obtained in the 12 months before the transplant, and at least one post-transplant evaluation were analyzed through the review of previous medical records. After organ transplantation, most patients receive immunosuppressive therapy with glucocorticoids, calcineurin inhibitors (cyclosporine A or tacrolimus), and either mycophenolate mofetil or azathioprine.

Informed consent was not required because this was a retrospective observational study; the biochemical assessment, DXA scans, and X-rays had been performed as part of routine clinical practice.

### Statistical Analysis

Clinical data and initial values of the measured variables in the study groups were compared using Student’s *t*-test for unpaired data. The two-tailed Student’s *t*-test and Mann–Whitney U-test were used to compare the differences between patient groups. Separate multiple linear regression models (method: stepwise) were used to assess independent predictors of vertebral fractures in lung and heart transplantation patients, while sex, age, body mass index (BMI), creatinine, vitamin D, LS-BMD, FN-BMD, TH-BMD, and history of osteoporotic fractures were included as independent variables in the models. For each model, the regression coefficients (b-coefficients) and their 95% confidence intervals were reported. A *p*-value < 0.05 was considered statistically significant.

All statistical tests were performed using SPSS 10.1 statistical software (SPSS 10.1).

## 3. Results

The clinical characteristics and biochemical and densitometric parameters at pre-transplantation, post-transplantation, and follow up in lung and heart recipients are shown in Table 1. There was a significant difference only for creatinine serum values between the two groups in baseline characteristics. The lung recipients group showed a significant increase in creatinine serum levels at the post-transplantation visit and at follow up. Moreover, BMD at the femoral sub-regions showed a significant decrease at post-transplantation (*p* < 0.01) and at follow up visits (*p* < 0.05). Additionally, BMD at the lumbar spine showed a mild and non-significant increase. In heart recipients, the creatinine serum levels declined at the pre-transplantation evaluation and remained stable thereafter, until follow up. BMD at the femoral sub-regions showed a progressive and significant reduction with respect to pre-transplantation values, even if minor with respect to lung recipients, at the post-transplantation visit. In these patients, LS-BMD also showed a mild reduction, which reached statistical significance at the follow up visit only.

**Figure 2:**
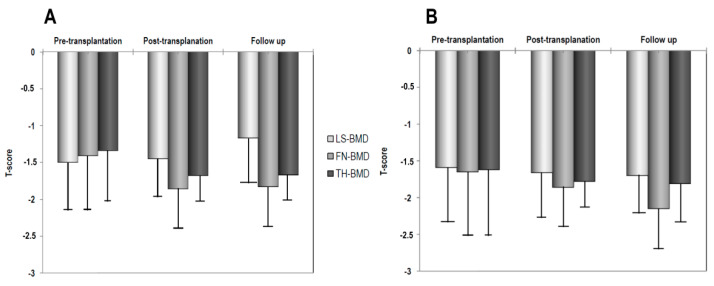
T-score values at pre-transplantation, post-transplantation, and follow up in lung (**A**) and heart recipients (**B**).

The values of BMD, expressed as T-scores, in lung and heart recipients over the study period are shown in Figure 2.

**Figure 3:**
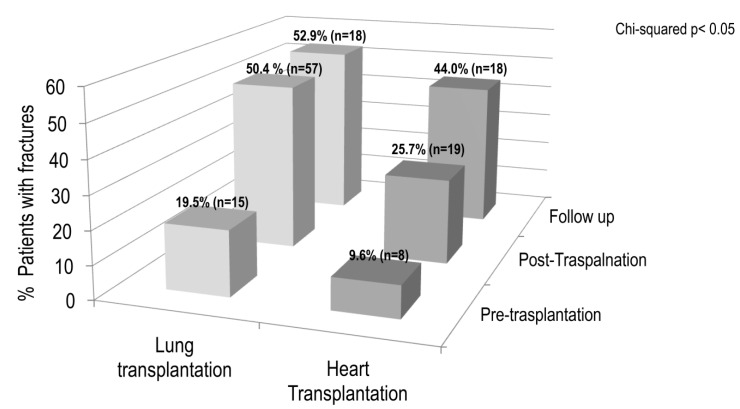
Presence of fragility fractures in lung and heart recipients at pre-transplantation, at post-transplantation, and at follow up.

The prevalence of pre-transplantation osteoporosis and osteopenia in lung and heart recipients was 30.1% and 52.7%, and 27.0% and 42.8%, respectively. Figure 3 shows the presence of fragility fractures in lung and heart recipients at pre-transplantation, at post-transplantation, and at follow up evaluation. The percentage of patients with fragility fractures was significantly higher (*p* < 0.05) in the lung recipient group compared to the heart recipients. However, the incidence of vertebral fractures markedly increased in the first period in both lung and heart recipients (from 19.5% to 50.4%, and from 9.6% to 25.7%, respectively). Thereafter, in the follow up period, the percentage of vertebral fractures remained almost unchanged in lung recipients, whereas it continued to increase in heart recipients.

**Figure 4:**
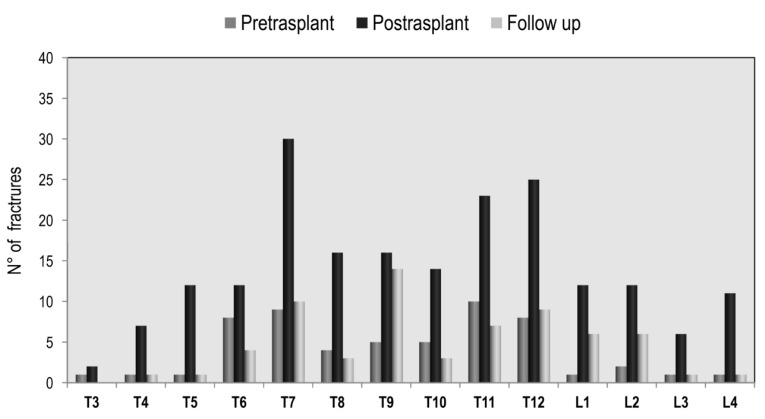
Distribution of vertebral fractures in lung transplantation.

**Figure 5:**
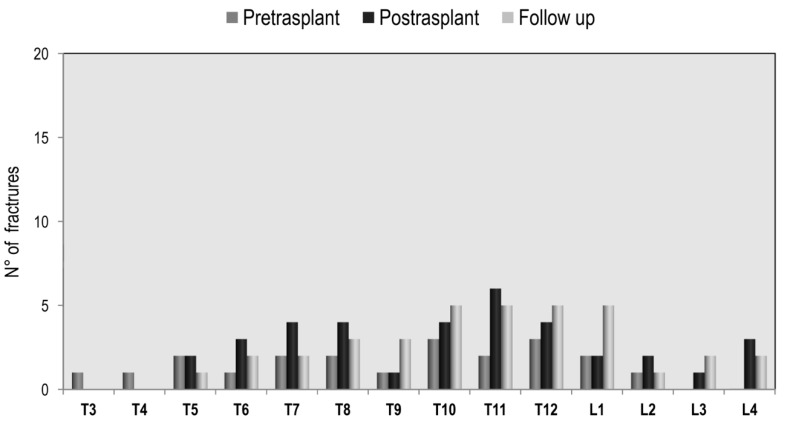
Distribution of vertebral fractures in heart transplantation.

The prevalence of vertebral fractures in lung recipients, by spinal location, is shown in Figure 4. The distribution of vertebral fractures along the spine at pre-transplantation evaluation showed a peak prevalence at T6 and T7. Another, if lower, peak prevalence was found at T11 and T12. At the post-transplantation assessment, the distribution of vertebral fractures showed a similar pattern. Similarly, in heart recipients, the distribution of vertebral fractures along the spine at pre-transplantation evaluation showed a peak prevalence at T7 and T8, and another peak at T10, T11, and T12 (Figure 5). Comparing the two study populations, a marked increase in the level of T7 fractures in lung recipients, with respect to heart recipients, is highlighted.

Multiple linear regression analyses of predictors of vertebral fractures in lung and heart transplantation recipients are reported in Table 2. The analyses were performed by including in the model sex, age, BMI, creatinine serum levels, 25OHVitamin D serum levels, LS-BMD, FN-BMD, TH-BMD, and history of osteoporotic fractures. In lung recipients, vertebral fractures were predicted by age, BMD at total hip, and history of fracture. In heart recipients, vertebral fractures were predicted only by history of fracture.

## 4. Discussion

This study, carried out on a large cohort of cardiac and lung transplant patients, shows that the presence and, above all, the burden of vertebral fractures are greater in the first period after transplantation. Our results are in agreement with previous studies reporting that patients with lung and heart diseases present with bone loss and increased risk of vertebral fracture within the first months after transplantation [13,14,15,16,17]. The causes of bone loss and fragility fractures in transplant patients are numerous. In fact, many risk factors, such as age, female gender, malnutrition, low BMI, past history of fractures, and use of immunosuppressant drugs, including glucocorticoids, lead to increased bone fragility in lung and heart transplant patients [3,11,14,18].

Moreover, this study confirms that lung transplant patients present with greater bone loss and higher fracture risk before lung transplantation, with respect to heart transplant recipients [4,19]. In our study, almost one fifth of the subjects had a vertebral fracture before lung transplantation, while less than 10% of heart patients had a history of vertebral fractures. A possible explanation for the higher percentage of fractures in lung transplantation subjects before transplantation could be the greater cumulative glucocorticoid dose, responsible for part of the bone loss through an uncoupling of bone remodelling, with a decrease in bone formation and an increase in bone resorption also occurring before transplantation [20]. This is also confirmed by the fact that the markers of bone formation were low during the pre-transplantation period and returned to normal after transplantation, whilst markers of bone resorption were increased before and after transplantation [20]. In addition, glucocorticoids cause inhibition of intestinal absorption and inhibit tubular reabsorption of calcium. The intestinal action of glucocorticoids is antagonistic of vitamin D, and there is a reduction in the expression of specific calcium channels in the duodenum. Additionally, the hypoxia, respiratory acidosis, tobacco use, and lower muscle mass that characterize end-stage lung disease contribute to the presence of fragility fractures [10,20]. The native lung disease has been hypothesized as contributing to bone abnormalities as well. In fact, a study by Vrieze et al. suggested a correlation between a low BMD and the severity of chronic obstructive pulmonary disease (COPD) [21]. Although in the literature there are controversial data regarding the association of osteoporotic fractures with severity in lung disease, some papers have reported that subjects with COPD present an increased fracture risk, independent of prior glucocorticoid therapy [22]. On the contrary, other studies have reported that disease severity is not significantly associated with fractures [23], but that vertebral fractures are associated with increasing age and that patients over 63 years of age are at higher risk. However, according to previous studies, vertebral fractures occur in the first period after transplantation, and may affect patients with either low or normal pre-transplant BMD [14]. A small number of prospective studies have evaluated the prevalence and incidence of vertebral fractures after lung transplantation [4,16,17,24,25,26,27]. All of these studies had limitations related to their relatively small sample sizes, and differences between studies may also result from the variety of underlying lung diseases and differences in the severity of these. However, our data, according to these latter studies [4,16,17,24,25,26,27], indicate that lung transplantation patients with the highest risk of vertebral fractures are those with low pre-transplantation BMD and a history of fragility fractures.

It is well established that significant bone loss after heart transplantation is common, and contributes to an increased risk of vertebral fractures [28,29]. Our study, like previous studies [30,31,32], showed an increase in vertebral fractures within the first period after heart transplantation, however in contrast to the outcome in lung transplant patients, the increase in the vertebral fracture incidence is maintained throughout the follow-up period. In fact, our data are in accordance with the study by Hofle et al. [33], which reported that long-term vertebral fracture incidence was greater than 40% five years after the transplant.

In the heart transplantation patients, the main factors associated with cardiac insufficiency, which may contribute to increased fragility fractures, are sarcopenia, reduced exercise, lack of sunlight exposure, low calcium intake, use of loop diuretics leading to urinary calcium loss, anticoagulant drug administration, smoking, and excessive alcohol intake, as well as other common osteoporosis factors [34]. Vitamin D deficiency and secondary hyperparathyroidism have been reported in patients with severe congestive heart failure. Magnesium depletion has also been noted, which may result from the use of diuretic therapy, as another putative contributor to osteoporosis in this setting [14]. An additional risk factor may be hypogonadism, caused by debilitating heart disease itself and treatment with calcineurin inhibitors [35]. Our data, in agreement with the study by Leidig-Bruckner et al. [32], showed a high rate of vertebral fractures over the first three years after transplantation.

A possible explanation for the different pattern of vertebral fracture risk between lung and heart recipients may be the severity of the pre-transplantation skeletal impairment.

Our study has some limitations. Firstly, the data were obtained through a review of medical records; secondly, bone mineral density by DXA was not performed in all cases; thirdly, the vertebral morphometry was carried out manually; and finally, hormonal status had not been routinely determined. Nevertheless, the strength of our study was in the large study sample and the long-term follow-up of the lung and heart transplantation patients.

In summary, our study confirmed a high vertebral fracture risk in lung and heart recipients, especially during the first period after transplantation. However, the risk of vertebral fractures may continue during the late follow-up period in heart recipients. The burden of vertebral fractures could potentially impact the clinical success of heart and lung transplants. In many of the lung and heart transplantation patients, bone disease was present before the transplant; therefore, more attention to skeletal risk factors in transplant candidates could reduce the frequency of fragility vertebral fractures that may occur after the transplant.

Therefore, our results support the recommendations for pre-transplant osteoporosis screening in patients undergoing lung transplants. Moreover, in heart transplantation patients, screening for vertebral fracture risk, at least in the first period after transplantation, is strongly recommended.

## Figures and Tables

**Table 1:** Clinical characteristics and biochemical and densitometric parameters at pre-transplantation, post-transplantation, and follow up in lung and heart recipients.

Parameters	Lung Recipients			Heart Recipients		
	Pre-Transplantation (*n* = 128)	Post-Transplantation (*n* = 113)	Follow Up (*n* = 34)	Pre-Trapnsplantation (*n* = 85)	Post-Transplantation (*n* = 73)	Follow Up (*n* = 41)
Age (y)	50.7 ± 11.8	52.9 ± 11.7	55.2 ± 13.5	51.1 ± 10.4	53.9 ± 10.3	56.9 ± 10.5
Weight (kg)	69.8 ± 15.1	70.9 ± 15.4	69.4 ± 11.7	73.7 ± 10.1	74.9 ± 11.9	72.9 ± 11.3
Height (cm)	168.1 ± 7.8 °	165.9 ± 16.1 *	167.7 ± 6.6 ^	172.8 ± 9.4	172.0 ± 8.1	169.3 ± 7.2
BMI (kg/m^2^)	24.7 ± 4.7	24.9 ± 4.4	24.6 ± 3.6	24.5 ± 2.9	25.4 ± 3.9	25.9 ± 3.9
Calcium (mg/dL)	9.2 ± 0.5	9.1 ± 0.6	9.3 ± 0.4	9.1 ± 0.7	9.4 ± 0.6	9.2 ± 0.5
Phosphorus (mg/dL)	3.5 ± 0.6	3.6 ± 0.7	3.4 ± 0.7	3.5 ± 0.6	3.3 ± 0.6	3.3 ± 0.5
Creatinine (mg/dL)	0.9 ± 0.2 °°	1.3 ± 0.5 **	1.3 ± 0.5 ^§§^	1.2 ± 0.3	1.3 ± 0.4	1.4 ± 0.5
Alkaline phosphatase (UI/L)	80.5 ± 48.3	72.8 ± 36.7	71.1 ± 29.6	90.5 ± 31.2	85.2 ± 39.1	73.2 ± 21.4
25OHD (ng/mL)	19.1 ± 11.8	21.6 ± 9.9	21.2 ± 9.3	22.4 ± 14.7	19.9 ± 14.6	23.8 ± 13.6
PTH (pg/mL)	42.7. ± 14.7	56.0 ± 46.1	57.8 ± 24.3	46.2 ± 35.2	76.9 ± 29.5	71.8 ± 39.6
LS-BMD (g/cm^2^)	1.011 ± 0.182	1.000 ± 0.194	1.025 ± 0.177 ^§,^^	0.988 ± 0.151	0.982 ± 0.155	0.964 ± 0.210 ^§^ ^
FN-BMD (g/cm^2^)	0.839 ± 0.167	0.765 ± 0.162 **	0.767 ± 0.121 ^§^	0.797 ± 0.140	0.779 ± 0.134	0.728 ± 0.135 ^
TH-BMD (g/cm^2^)	0.863 ± 0.172	0.818 ± 0.157 **	0.819 ± 0.138 ^§^	0.838 ± 0.112	0.811 ± 0.126 *	0.798 ± 0.102 ^

Pre-transplantation lung recipient vs. Pre-transplantation heart recipient ° *p* < 0.05; °° *p* < 0.01, Pre-transplantation vs. Post-transplantation * *p* < 0.05; **p < 0.01, Pre-transplantation vs. Follow up ^§^ *p* < 0.05; ^§§^ *p* < 0.01, Post-transplantation vs. Follow up ^ *p* < 0.05.

**Table 2:** Multiple linear regression analysis of predictors for the vertebral fracture in lung and heart transplantation recipients.

Variable	Undestandardized Coefficient, b	95%CI	*p*
Lung			
Vertebral fracture			
Age	0.018	0.006 to 0.029	0.003
TH-BMD	−1.421	−2.284 to −0.558	0.002
History of fracture	0.382	0.003 to 0.760	0.048
Heart			
Vertebral fracture			
History of fracture	0.867	0.510 to 1.224	<0.01

Whole set of variables included into the model heart: sex, age, BMI, creatinine, vitamin D, LS-BMD, FN-BMD, TH-BMD, history of osteoporotic fractures.
